# Efficacy and safety of perampanel as the first medication for children with newly diagnosed epilepsy: a real-world single-center prospective observational study

**DOI:** 10.3389/fped.2026.1767763

**Published:** 2026-05-28

**Authors:** Wenmin Huang, Dezhi Cao

**Affiliations:** 1 Shenzhen Children's Hospital, Shantou University Medical College, Shenzhen, China; 2 Department of Neurology, Shenzhen Children’s Hospital, Shenzhen, China

**Keywords:** anti-seizure medications, children's epilepsy, monotherapy, perampanel, self-limited epilepsy with centrotemporal spikes

## Abstract

**Introduction:**

Perampanel (PER) is an antiseizure medication (ASM) with a unique mechanism of action. This study aimed to evaluate the clinical efficacy and safety of PER as the initial monotherapy in pediatric epilepsy and investigate the factors linked to its efficacy.

**Methods:**

Between August 2021 and August 2023, we conducted a single–center, prospective observational study at Shenzhen Children's Hospital on children with epilepsy treated with PER monotherapy as the initial ASM. The primary outcome indicators were the seizure–free, response, and medication retention rates at 6 and 12 months. Additionally, adverse events (AEs) were also recorded.

**Results:**

In this study, 137 patients with epilepsy were included. Among these patients, two patients were lost to follow-up (attrition rate, 1.5%), seven patients discontinued taking PER due to intolerable AEs (discontinuation rate, 5.1%), and finally, 128 patients were retained (63 males, 65 females; mean age, 7.91 ± 2.53 years). The median duration of epilepsy history prior to PER monotherapy was 0.18 years (interquartile range: 0.04–0.59 years), and the mean initial dosage of PER was 2.3 ± 1.2 mg/day. Among the 128 patients, 42 were diagnosed with self-limited epilepsy with centrotemporal spikes (SeLECTS). Thirty-three patients diagnosed with SeLECTS were seizure-free during the 12-month follow-up period after PER administration (seizure-free rate, 78.6%). The response rates of PER treatment at different time points were 92.9% (6 months) and 90.6% (12 months). Additionally, with PER treatment, the seizure-free rate varied over time, with 75.0% (96/128) and 64.1% (82/128) of patients achieving seizure freedom at the 6-month and 12-month follow-ups, respectively. At the follow-up visit, 34 patients experienced AEs. The average AEs rate was 24.8%. Common AEs were dizziness (20 patients), drowsiness (9 patients), and irritability (7 patients), most of which were mild and transient; patients gradually tolerated and were retained by slow titration or adjustment of the maintenance dose of PER. Sixty-four patients who received 4 mg/day of PER had the highest seizure-free rate (70.3%).

**Discussion:**

In this single-arm observational study, PER monotherapy proved effective and safe for children with epilepsy. The seizure-free rate at 12 months stood at 64.1% (82/128), showing higher rates in the SeLECTS subgroup (78.6%, 33/42) compared to the non-SeLECTS subgroup (56.9%, 49/86).

## 1 Introduction

Epilepsy is a common neurological disorder, with approximately 75% of cases occurring in childhood (1). The prevalence of epilepsy in the pediatric population is estimated to be between 0.5% and 1% (2). Globally, approximately 50 million individuals are affected by epilepsy (3), with nearly 10 million cases reported in China (4, 5). Antiseizure medications (ASMs) are the primary treatment for epilepsy, effectively controlling seizures in approximately 63% of patients. Rational monotherapy can render approximately 47% of patients seizure-free, thereby improving their quality of life and reducing the number of children with refractory epilepsy (6)^.^ Hence, the initial selection of ASMs is crucial in clinical practice.

Perampanel (PER) is a novel third-generation ASM that functions as a non-competitive antagonist of the *α*-amino-3-hydroxy-5-methyl-4-isoxazolepropionic acid (AMPA) type glutamate receptor, inhibiting neurotransmitter delivery to manage seizures. In 2019, PER received initial approval for marketing in China as an adjunctive therapy for children aged over 12 years with focal seizures, with or without secondary generalized seizures. Subsequently, in July 2021, PER was approved for monotherapy to treat children with focal epilepsy aged over 4 years. At present, PER as an adjunct therapy remains the primary approach for epilepsy in China, with substantial real-world data supporting its efficacy in pediatric patients with epilepsy (7–9). However, clinical research on PER as the initial treatment is still limited. This study, involving 137 epilepsy patients at the Department of Neurology and Epilepsy Center of Shenzhen Children's Hospital, aimed to assess the clinical effectiveness and safety of PER as the first-line monotherapy for epilepsy treatment.

## 2 Materials and methods

### 2.1 Study design

In this 12-month follow-up prospective single-center observational study, we evaluated the effectiveness and safety of PER as the primary monotherapy in pediatric epilepsy patients. Inclusion criteria comprised: (1) newly diagnosed epilepsy per the International League Against Epilepsy (ILAE) criteria at Shenzhen Children's Hospital between August 2021 and August 2023; (2) age between 4 and 18 years; (3) at least two seizures in the preceding 6 months, with one occurring in the last 3 months; (4) patients prescribed PER as the primary monotherapy. Exclusion criteria included: (1) patients with psychiatric disorders or impulsive behaviors (based on DSM-5 criteria); (2) comorbid severe organ dysfunction; (3) involvement in another drug or medical device clinical trial within 60 days before enrollment; (4) patients with progressive neurodegenerative conditions (e.g., mitochondrial disorders, neuronal ceroid lipofuscinosis, progressive myoclonus epilepsies). Developmental and epileptic encephalopathies lacking a clear progressive neurodegenerative course (e.g., Lennox–Gastaut syndrome, Dravet syndrome) were not excluded if they met other inclusion criteria: (5) used traditional Chinese medicine for epilepsy, or (6) had non-epileptic seizures. Seizure types were categorized following the 2017 ILAE Classification of Seizure Types (10). Epilepsy syndromes were classified per the 2022 ILAE Classification and Definition of Epilepsy Syndromes (11). All patients underwent routine diagnostic evaluations at baseline, including long-term video electroencephalography (EEG) with sleep-stage recording and brain magnetic resonance imaging (MRI) at 3.0 T. These assessments were used to confirm the diagnosis and classify epilepsy syndromes according to ILAE criteria. The study received approval from the Ethics Committee of Shenzhen Children's Hospital (ID: 202104903).

All patients received PER once daily at bedtime. The dosage was adjusted by 1 mg per week for individuals weighing less than 30 kg and by 2 mg per week for those weighing 30 kg or more until reaching the maintenance dosage (4 mg/day or more). The PER dosage was increased by 1–2 mg at intervals of 1–4 weeks to achieve a recommended maintenance dosage of 4 mg/day or more, with a maximum of 12 mg/day. Patients were categorized into subgroups based on the type of seizures and epilepsy syndrome: self-limited epilepsy with centrotemporal spikes (SeLECTS) and non-SeLECTS groups. Since SeLECTS is a prevalent pediatric syndrome in clinical settings, a subgroup analysis was conducted to evaluate the effectiveness of PER in this specific population. For patients diagnosed with SeLECTS, the decision to initiate PER monotherapy was based on predefined clinical indications derived from StatPearls guidelines. Treatment was considered if patients met any of the following criteria: (1) frequent seizures; (2) severe seizures; (3) daytime seizures; (4) seizures associated with language decline, neurocognitive decline, or learning disorders; (5) generalized seizures (focal to bilateral tonic-clonic) (12); (6) parental preference for active treatment due to anxiety about recurrent seizures (13). Patients were further classified into seizure-free and seizure groups based on the efficacy of PER monotherapy.

### 2.2 Baseline data, effectiveness, and safety assessment

This study examined factors influencing the efficacy of PER monotherapy in patients, such as sex, weight, age, duration of epilepsy history, number of seizures during the 3 months before enrollment, type of seizure, etiology, and initial and maintenance PER doses. Seizure frequency was classified as seizure-free, improvement, treatment inefficacy, or seizure worsening. Seizure-free status was defined as no epileptic seizures during the 12-month follow-up. Improvement indicated a ∼50% decrease in seizure frequency compared to baseline. Treatment inefficacy meant <50% decrease, while seizure worsening meant a 10% increase in seizures compared to the baseline. Seizure frequency and adverse events (AEs) in children were monitored during outpatient visits. At each follow-up visit, the current dose of PER, seizure frequency, and AEs were recorded, including timing, severity, and outcomes. AEs leading to treatment discontinuation and the time of discontinuation were also documented. All AEs were graded per Common Terminology Criteria for Adverse Events (CTCAE) version 5.0 (14) as Grade 1–2 (mild/moderate) and Grade 3 (severe). Factors associated with AE were also investigated.

### 2.3 Data analysis

During our follow-up period, the investigator collected data from routine follow-up visits, including titration and maintenance doses of PER, as well as the type and number of seizures in the 3 months before enrollment. Quantitative data were presented as mean ± standard deviation or median, while count data were expressed as *n* (%). The retention rate, excluding patients lost to follow-up, indicating the percentage of patients continuing ASM with PER at 6 and 12 months, was calculated. The normality of continuous variables, such as the initial and maintenance doses of PER, was assessed using the Shapiro–Wilk test. A *p*-value > 0.05 indicated a normal distribution. Independent sample t-tests were used for normally distributed data, and the Mann–Whitney U test was applied for non-normally distributed data. Categorical variables, including sex, type of seizure, and etiology, were analyzed using the chi-squared test or Fisher's exact test. All statistical tests were two-tailed, with a significance level set at *α* = 0.05.

## 3 Results

### 3.1 Patient characteristics

**Figure 1:**
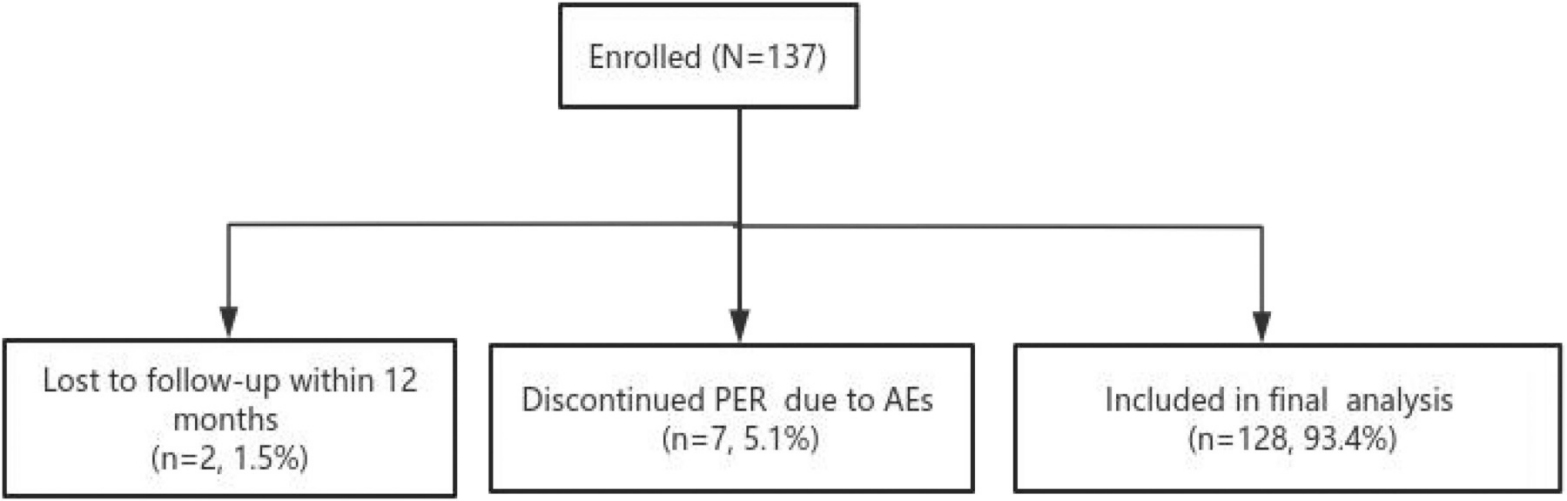
Patient flow diaram. A total of 137 patients were enrolled. Two patients (1.5%) were lost to follow-up within 12 months. Seven patients (5.1%) discontinued perampanel due to adverse events. The remaining 128 patients completed the 12-month follow-up and were included in the final analysis.

Among 137 patients initially included, two were excluded due to loss of follow-up within 12 months. Consequently, 135 patients were retained for analysis. Among them, seven patients (5.1%) discontinued PER within 12 months due to severe AEs (Figure 1). The retention rates at 6 and 12 months were 96.3% (130/135) and 94.8% (128/135), respectively (Table 1). Patients who discontinued PER or were lost to follow-up were excluded, leaving 128 patients (Table 2), comprising 63 males and 65 females, with an average initial PER dose of 2.3 ± 1.2 mg.

**Table 1:** Efficacy and tolerability outcomes of perampanel monotherapy at 6 and 12 months.

Outcome	6 months	12 months
Seizure-free rate	75.0% (96/128)	64.1% (82/128)
Response rate^a^	92.9% (119/128)	90.6% (116/128)
Adverse events	23.4% (32/137)	24.8% (34/137)
Retention rate^b^	96.3% (130/135)	94.8% (128/135)

a Response rate: >50% reduction in seizure frequency.

b Retention rates were calculated after excluding two patients lost to follow-up.

**Table 2:** Comparison of baseline data of different efficacy groups after 12 months (*n* = 128).

Categories	Total (*n* = 128)	Seizure-free group (*n* = 82)	Seizure group (*n* = 46)	*p*-value
Gender, n(%)				0.895
Male	63 (49.2)	40 (48.8)	23 (50.0)	
Female	65 (50.8)	42 (51.2)	23 (50.0)	
Age, n(%)				
4–7y	65 (50.8)	43 (52.4)	22 (47.8)	0.616
8–18y	63 (49.2)	39 (47.6)	24 (52.2)	
Type of seizure, n(%)				1.00
Focal-onset seizure (with or without generalized seizures)	123 (96.1)	80 (97.6)	43 (93.4)	
Generalized-onset seizure				
—Generalized tonic-clonic	3 (2.3)	2 (2.4)	1 (2.2)	
—Myoclonic	1 (0.8)	0 (0)	1 (2.2)	
—Absence	1 (0.8)	0 (0)	1 (2.2)	
Etiology, n(%)				0.579
Structural	8 (6.3)	5 (6.1)	3 (6.5)	
— hippocampal sclerosis	2 (1.6)	1 (0.8)	1 (0.8)	
— malformations of cortical development	5 (3.9)	3 (2.3)	2 (1.7)	
— encephalomalacia	1 (0.8)	1 (0.8)	0 (0)	
Non-structural	120 (93.7)	77 (93.9)	43 (93.5)	
Genetic	6 (4.7)	5 (3.9)	1 (0.8)	
Both genetic and structural	2 (1.6)	2 (1.6)	0 (0)	
Metabolic	0 (0.0)	0 (0.0)	0 (0.0)	
Immune	0 (0.0)	0 (0.0)	0 (0.0)	
Infectious	0 (0.0)	0 (0.0)	0 (0.0)	
Unknown	112 (87.4)	70 (88.4)	42 (92.7)	
Epilepsy Syndrome, n(%)				0.017
SeLECTS	42 (32.8)	33 (40.2)	9 (19.6)	
Non-SeLECTS	86 (67.2)	49 (59.8)	37 (80.4)	
Dose of PER				0.702
Initial dose(mg/day), mean ± SD	2.3 ± 1.2	2.4 ± 1.3	2.3 ± 0.8	
Maintenance dose, n(%)				0.252
＜4 mg/day	17 (13.3)	13 (15.9)	4 (8.7)	
≥4 mg/day	111 (86.7)	69 (84.1)	42 (91.3)	
Number of seizures during the 3 months before enrollment, median (IQR)	3.00 (0.99–9.00)	3.00 (1.14–9.00)	3.00 (0.99–9.00)	0.776
Duration of epilepsy history(y), median (IQR)	0.18 (0.04–0.59)	0.23 (0.04–0.59)	0.17 (0.07–0.50)	0.823

SD, standard deviation; PER, perampanel; IQR, interquartile range.

The patients in this study experienced various types of seizures, including focal, myoclonic, absent, and generalized tonic-clonic seizures. Of these patients, 123 experienced focal-onset seizures with or without generalized seizures, while 5 experienced generalized onset seizures. There was no statistically significant difference in the treatment outcomes between the two groups (*p* = 1.000). Among the enrolled patients, 8 were diagnosed with structural epilepsy, 6 were diagnosed with genetic epilepsy, 2 with mixed etiology involving both structural and genetic factors, and 112 were diagnosed with epilepsy of unknown etiology. The cohort included 42 patients (32.8%) with SeLECTS and 86 (67.2%) without SeLECTS. Detailed comparisons of the baseline characteristics are presented in Table 1. Structural etiologies included hippocampal sclerosis (*n* = 2), malformations of cortical development (*n* = 5), and encephalomalacia (*n* = 1). Among the eight patients with genetic component, variants were found in MECP2 (*n* = 2), KCNQ3 (*n* = 1), KDM6A (*n* = 1), and AFF3 (*n* = 1). One patient carried a variant of C12orf65, and another had large chromosomal alterations with a pathogenic duplication at 18q11.1-q23, and a third exhibited a large deletion at Xq23. Among patients with SeLECTS, seizure-free, condition improvement, treatment inefficacy, and seizure worsening were 33, 7, 1, and 1, respectively.

### 3.2 Dose of PER

In the 137 patients, the median duration of epilepsy history was 0.18 years (interquartile range 0.04–0.59 years). PER monotherapy typically starts at a dose of 1–2 mg once daily and is given at bedtime. The titration schedule is personalized, with the targeted dose adjusted based on clinical response and tolerability. The average maintenance dose for PER monotherapy over 12 months is 4.41 ± 1.16 mg/day (equivalent to 0.16 ± 0.10 mg/kg/day).

### 3.3 Efficacy

In our investigation, 82 patients achieved seizure freedom and 34 showed improvement in their condition after 12 months (Table 3). Notably, PER treatment was ineffective in 7 patients and exacerbated symptoms in 5 patients. The latter group received additional ASM as part of their subsequent treatment. Detailed clinical profiles of these five patients, including etiology, seizure types, and PER dosages, are provided in the Supplementary Table. The response rate to PER treatment at the 12-month mark was 90.6%. Before enrollment, the median number of seizures over three months was 3.00 (IQR: 0.99–9.00). By the 6-month follow-up, the median number of seizures decreased to 0 (IQR: 0–1), with 75% (96/128) of patients achieving complete seizure freedom. At the 12-month follow-up, the median number of seizures remained at 0 (IQR: 0–1), with a seizure-free rate of 64.1% (82/128). To evaluate the impact of SeLECTS enrichment on overall efficacy, we conducted a sensitivity analysis excluding patients with SeLECTS. In the non-SeLECTS subgroup (*n* = 86), the 12-month seizure-free rate was 56.9% (49/86), and the response rate was 88.3% (76/86). The incidence of AEs among the study participants was 24.8% (34/137).

**Table 3:** Efficacy outcomes at 12 months (*n* = 128).

Outcome	n (%)
Seizure free, *n* (%)	82 (64.1)
Improvement, *n* (%)	34 (26.6)
Treatment inefficacy, *n* (%)	7 (5.5)
Seizure worsening, *n* (%)	5 (3.8)

According to the classification of epileptic syndromes, the enrolled patients were categorized into SeLECTS and non-SeLECTS groups. In the SeLECTS group, 78.6% (33/42) of patients were seizure-free, and 95.2% (40/42) responded positively. For the non-SeLECTS group, 56.9% (49/86) of patients were seizure-free, and 88.3% (76/86) responded positively (Table 4). No significant difference in the initial PER dose between the two groups were observed (*p* = 0.929). The seizure-free rate in patients with SeLECTS was higher than in the non-SeLECTS groups. There was no statistical difference in AEs between the groups (*p* = 0.147)**.**

**Table 4:** Baseline characteristics and clinical outcomes at 12 months in SeLECTS vs. non-SeLECTS groups (*n* = 128).

Characteristic	Total (*n* = 128)	SeLECTS (*n* = 42)	non-SeLECTS (*n* = 86)
Seizure-free rate at 12-month, n(%)	64.1	78.6	56.9
Response rate at 12-month, n(%)	90.6	95.2	88.3
Number of seizures during the 3 months before enrollment, median (IQR)	3.00 (0.99–9.00)	5.00 (1.50–22.50)	2.58 (0.99–6.00)
Type of seizure, n(%)			
Focal-onset seizure (with or without generalized seizures)	123 (96.1)	42 (100)	81 (94.1)
Generalized-onset seizure			
—Generalized tonic-clonic	3 (2.4)	0 (0.0)	3 (3.5)
—Myoclonic	1 (0.8)	0 (0.0)	1 (1.2)
—Absence	1 (0.8)	0 (0.0)	1 (1.2)

### 3.4 Safety and tolerability

Among the 137 children enrolled in the study, AEs were observed in 34 patients (24.8%). The majority of AEs were mild and well-tolerated. Among the participants, 11 patients (8.0%) experienced Grade 1 AEs according to the CTCAE, while 16 patients (11.7%) had grade 2 AEs. Seven patients (5.1%) discontinued the PER monotherapy early due to Grade 3 AEs related to the drug. The documented AEs included irritability (*n* = 1), drowsiness (*n* = 1), allergy (*n* = 1), abdominal pain (*n* = 1), dizziness (*n* = 2), and memory decline (*n* = 1). The remaining patients gradually developed tolerance to the prescribed dose following a slow titration process (see Table 5). An analysis of the factors associated with AEs revealed no statistically significant correlations between the occurrence of AEs and age, sex, or PER dosage (*p* > 0.05) (see Table 6).

**Table 5:** Adverse events (AEs) during the 12-month follow-up.

Classification	Total
Adverse events (*n* = 34)^a^	
Dizziness, *n* (%)	20 (58.8)
Drowsiness, *n* (%)	9 (26.4)
Irritability, *n* (%)	7 (20.5)
Decreased Reaction, *n* (%)	2 (5.8)
Memory Decline, *n* (%)	2 (5.8)
Abdominal Pain, *n* (%)	1 (2.9)
Rash, *n* (%)	1 (2.9)
Diplopia, *n* (%)	1 (2.9)
Xerostomia, *n* (%)	1 (2.9)
Severity of adverse events(*n* = 34)	
Grade 1, *n* (%)	11 (32.4)
Grade 2, *n* (%)	16 (47.1)
Grade 3, *n* (%)	7 (20.5)

a during the 12 months, 27 patients experienced 1 type of AEs, 6 patients experienced 2 types of AEs, 1 patients experienced 3 types of AEs. All seven patients who discontinued PER due to Grade 3 AEs are included in the AE type counts presented in this table.

**Table 6:** Clinical characteristics of patients with and without AEs (*n* = 137).

Characteristic	No AE group (*n* = 103)	AEs group (*n* = 34)	*p*-value
Age (years), mean ± SD	7.94 ± 2.54	8.43 ± 3.11	0.364
Gender, *n* (%)			0.067
Male	48 (46.6)	22 (64.7)	
Female	55 (53.4)	12 (35.3)	
Weight (kg), mean ± SD	28.61 ± 11.60	30.1 ± 12.05	0.541
Initial dose (mg/day), mean ± SD	2.39 ± 1.24	2.15 ± 0.79	0.288
Maintenance dose (mg/day), mean ± SD	4.34 ± 1.25	4.24 ± 1.21	0.666

### 3.5 Retention rate

Of the 137 patients in the study, seven stopped PER due to AEs, and two were lost to follow-up. After excluding the two patients lost to follow-up, drug retention rates at 6 and 12 months were 130/135 (96.3%) and 128/135 (94.8%), respectively.

### 3.6 Factors influencing efficacy

At 12 months, there were no significant differences between the seizure-free and seizure groups in terms of sex, mean age, duration of epilepsy history, type of epilepsy, baseline seizure frequency, or mean maintenance dose of PER (Table 1). No significant difference was observed between the 4 and 7-and 8–18 years age groups (*p* = 0.616). However, statistically significant difference was observed in the efficacy between the SeLECTS and non-SeLECTS groups (Table 1). Multivariate logistic regression analysis showed that SeLECTS (95% CI: 1.22–6.68, *p* = 0.016) was independently associated with seizure freedom at 12 months. The baseline seizure frequency, age, and sex were not significant predictors (Table 7).

**Table 7:** Multivariate logistic regression analysis of factors associated with seizure freedom at 12 months(*n* = 128).

Variable	Adjusted OR	95% CI	*p*-value
Epilepsy Syndrome	2.88	1.22–6.86	0.016
Number of seizures during the 3 months before enrollment	1.00	0.60–6.93	0.253
Age	1.03	0.89–1.20	0.693
Sex	0.85	0.40–1.82	0.683
Maintenance dose	0.99	0.99–1.02	0.413

OR, odds ratio; CI, confidence interval. Epilepsy Syndrome: SeLECTS vs. Non-SeLECTS; Age: 4–7y vs. 8–18y. Sex: male vs. female; Maintenance dose: ≥ 4 mg/day vs. < 4 mg/day.

## 4 Discussion

At present, high-quality evidence supports the use of PER in the treatment of childhood epilepsy (15), with add-on therapy as the main approach in China (16, 17). Limited studies have investigated the optimal monotherapy with PER in children, confirming its efficacy as the preferred treatment for childhood epilepsy (9, 16, 18). In a single-center observational study on PER monotherapy in Wuhan, 71 children with epilepsy participated, showing retention rates of 84.1% at 6 months and 78.4% at 12 months, with overall seizure-free rates of 83.5% and 82.9% (19). A prospective multicenter study in the United States explored PER as the first-line monotherapy for childhood epilepsy, reporting a 12-month retention rate of 77.8% and a seizure-free rate of 44.4% (20). By contrast, our study demonstrated higher retention (96.3% at 6 months and 94.8% at 12 months) and response rates (92.9% at 6 months and 90.6% at 12 months). These differences may be attributed to factors such as epilepsy syndrome and dosing strategies. Notably, our study population had a significantly younger mean age (7.91 years), potentially impacting the pharmacodynamic properties of PER due to differences in AMPA receptor subunit composition in childhood compared to adolescence (21). However, this hypothesis was not directly tested in our study. Furthermore, SeLECTS cases represented 32.8% (42/128) of our study population, a higher prevalence than in the general epilepsy population. This enrichment likely contributed to the overall seizure-free rate, as SeLECTS is known to have a self-limited course. To mitigate potential bias, we conducted a sensitivity analysis excluding patients with SeLECTS. The efficacy observed in the non-SeLECTS subgroup (56.9% seizure-free rate at 12 months) provides a more generalizable estimate for newly diagnosed childhood epilepsy. The combined effects of age-related and syndrome-specific factors explain the divergent outcomes between the studies. Most patients in our study experienced fewer seizures in the 3 months before enrollment and received a lower initial PER dose, potentially enhancing tolerability and retention rates.

In this study, we examined the factors affecting the preference for PER as the primary therapy for epilepsy in children over 4 years old. Our analysis revealed no notable variations in treatment outcomes based on gender, age, duration of epilepsy, seizure type, seizure frequency in the three months preceding enrollment, or PER dosage. Patients diagnosed with SeLECTS exhibited superior response rates and a higher likelihood of becoming seizure-free.

In several small-sample clinical studies and case reports, PER has demonstrated promising results in various types of childhood epilepsy, including epilepsy syndromes such as Dravet syndrome (22), Lennox–Gastaut syndrome (23), and some refractory epilepsies (24). Some studies indicate the efficacy of adjunctive therapy for patients with SeLECTS and sustained electro-epileptic status epilepticus during sleep (8). However, reports on the therapeutic effects of PER in patients with SeLECTS are scarce. Zhang et al. (8) revealed that patients with SeLECTS responded to six months of PER adjunctive therapy with a seizure-free rate of 75%. In this study, 42 patients diagnosed with SeLECTS were enrolled. Pediatric patients with SeLECTS exhibited higher seizure-free survival and response rates compared to those without SeLECTS, consistent with previous findings (8, 16, 25). It should be acknowledged that current international guidelines typically recommend levetiracetam or lamotrigine as the first-line monotherapy for SeLECTS when treatment is indicated (13), and the evidence base for PER in this setting remains limited, consisting primarily of observational studies. However, emerging real-world evidence suggests that PER monotherapy may be a reasonable alternative. A retrospective study of 86 children with newly diagnosed SeLECTS reported a 12-month seizure-free rate of 79.07% and a retention rate of 98.83% with PER monotherapy (26). Another study of 62 children with newly diagnosed focal epilepsy found that the SeLECTS subgroup had an efficacy rate exceeding 80% (27). For these patients who met treatment indications in this study, PER achieved a 78.6% seizure-free rate at 12 months, which is comparable to or higher than reported rates for conventional first-line agents (28).Given PER's unique mechanism of action as a selective non-competitive AMPA receptor antagonist and its demonstrated efficacy, tolerability, and cognitive safety in SeLECTS, PER may be considered a reasonable monotherapy option—particularly for patients who are intolerant to conventional agents or have behavioural concerns. PER suppresses glutamate-mediated hyperexcitability during non-rapid eye movement sleep, coinciding with the peak of epileptiform activity in SeLECTS (29). Although this study lacked polysomnography data to confirm sleep-stage specificity, this mechanism warrants validation in future research. Therefore, larger comparative studies with levetiracetam or lamotrigine are needed to determine whether PER offers any efficacy or tolerability advantages over current standard-of-care options.

**Figure 2:**
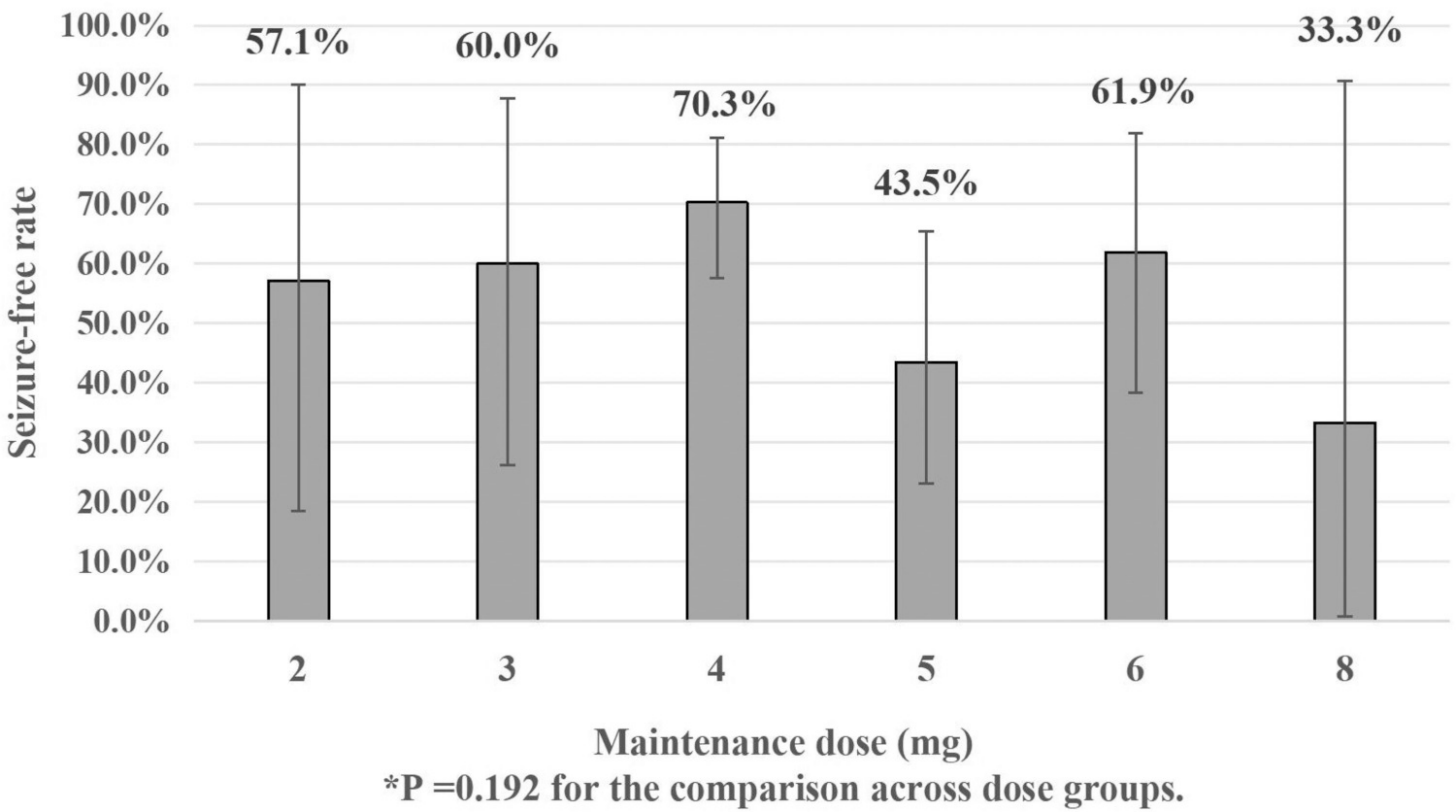
Seizure-freedom rates in patients taking 2 mg/day, 3 mg/day, 4 mg/day, 5 mg/day, 6 mg/day and 8 mg/day PER. Error bars represent 95% confidence intervals (CI). Dose groups (n below *x*-axis): 2 mg (7), 3 mg (10), 4 mg (64), 5 mg (23), 6 mg (21), 8 mg (3). Seizure-free rates (95% CI): 57.1% (18.4%–90.1%), 60.0% (26.2%–87.8%), 70.3% (57.6%–81.1%), 43.5% (23.2%–65.5%), 61.9% (38.4%–81.9%), 33.3% (0.8%–90.6%), respectively. Between-group difference: not significant (Fisher's exact test, *p = 0.192).

Compared to patients receiving less than 4 mg PER daily, those treated with at least 4 mg/day PER in this study exhibited a lower seizure-free rate, but the difference was not statistically significant (*p* = 0.252). Among the 64 patients maintained on 4 mg/day PER, the seizure-free rate was the highest (Figure 2). By contrast, patients maintained on 5 or 6 mg/day of PER showed a lower seizure-free rate than those on 2 or 3 mg/day. Current clinical evidence on the relationship between PER maintenance dose and therapeutic efficacy remains inconsistent. A retrospective study from the Philippines reported that children aged 1–18 years with epilepsy treated with ≥4 mg/day PER had a higher response rate than those receiving <4 mg/day (30). A pooled analysis of three randomized controlled trials showed a dose-dependent effect when PER was used as adjunctive therapy at doses of 4, 6, and 8 mg/day. Among patients with refractory focal-onset seizures, the 50% and 75% responder rates were highest in the 8 mg/day group, surpassing those in the 6 mg/day and 4 mg/day groups. Meanwhile, a single-center prospective study in Chinese children with newly diagnosed focal epilepsy found that compared with patients receiving 4 mg or 8 mg PER monotherapy, those receving 6 mg/day PER monotherapy showed no significant numerical or statistical improvement in the seizure-free rate at 6 and 12 months (*p* = 0.907 and 0.913, respectively) (31). These findings align more closely with observations from Study 342 (9), where patients with newly diagnosed or recurrent focal epilepsy received 32 weeks of PER monotherapy starting at 4 mg/day. In that study, patients on 4 mg/day of PER achieved a higher seizure-free rate than those on 8 mg/day of PER (63% vs. 38%, respectively), suggesting that patients requiring higher PER doses inherently have more difficult-to-control seizures. Confounding by indication must be considered when interpreting dose-related findings, as the lower seizure-free rates in higher dose groups most likely reflect baseline differences in seizure severity. Therefore, the optimal maintenance dose of PER should be individualized based on clinical response and patient tolerability. It is important to note that the number of patients in the high-dose groups, particularly the 8 mg group (*n* = 3), was relatively small. Consequently, comparisons involving these dose groups should be considered for hypothesis generation. By contrast, the finding that a maintenance dose of ≥4 mg/day is independently associated with seizure freedom is based on a substantially larger patient cohort and represents the primary dose-related conclusion of this study.

Previous studies have shown that the primary AEs linked to PER are drowsiness, irritability, and nasal discomfort (32, 33). In our research, patients exhibited excellent PER tolerance, with only seven discontinuing treatment due to severe AEs. Among the 34 patients (24.8%) who experienced AEs, 27 encountered mild or moderate AEs. Twenty-seven patients gradually acclimated to the AEs and persisted with PER usage, showing improved tolerance through slow titration or dose adjustments. A total of seven patients (5.1%) ceased treatment due to severe AEs (Grade 3). Common AEs observed in our study included dizziness (*n* = 20), drowsiness (*n* = 9), and irritability (*n* = 7), consistent with prior findings. A small number of patients reported memory decline (*n* = 2). Previous studies have documented AE rates between 16.5% and 22.9% in pediatric cohorts treated with PER (16, 34). Our study revealed an overall AE incidence of 24.8%, surpassing rates in previous pediatric PER studies. However, the low discontinuation rate (5.1%) highlights the favorable safety profile of PER as a first-line monotherapy for pediatric epilepsy. No significant correlations were identified between AE occurrence and age, sex, or PER dosage.

According to the etiological classification, the study included patients with genetic, structural, or unknown etiologies. Eight patients had genetic factors, with two showing mutations in MECP2. Both patients had neurodevelopmental impairments and focal epilepsy. Patient A had intellectual developmental delay, while Patient B showed symptoms of autism spectrum disorder. Both groups experienced reduced seizure frequency. At the 12-month follow-up, improvements in the patients’ conditions and seizure freedom were observed. Owing to the limited number of cases (*n* = 2) in this study, definitive conclusions on the efficacy and mechanism of PER in patients with genetic etiologies could not be made, necessitating further investigation.

This study had several limitations. Firstly, the sample size was inadequate, especially for the structural and genetic epilepsy subgroups. Moreover, there was a deficiency of infectious epilepsy cases and metabolic and immune epilepsy cases, as these instances were absent among the consecutively screened patients meeting the inclusion criteria. While the study's findings imply potential efficacy in these populations, the limited subgroup sizes constrained the statistical power to identify clinically significant differences. Secondly, as an observational study in a real-world setting, the absence of a placebo control group hindered definitively attributing seizure reduction solely to PER. Thirdly, the 12-month follow-up period proved insufficient for assessing long-term outcomes, such as cognitive development or adverse events that may emerge over time. Therefore, to confirm the initial observations, particularly regarding specific genetic variants, a higher-quality perspective with a larger sample size and extended observation period involving a control group is essential. This would allow comprehensively investigating the efficacy and safety of PER as a preferred monotherapy for pediatric epilepsy and its effectiveness in sleep-related seizures such as SeLECTS. Additionally, this study excluded patients with underlying psychiatric disorders, potentially leading to an underestimation of behavioral AEs. The study also lacked detailed EEG and neuroimaging data of epilepsy patients.

## 5 Conclusion

In this prospective, single-center observational clinical study, we assessed the effectiveness and safety of PER as the initial monotherapy for pediatric epilepsy. PER monotherapy achieved an overall response rate of 90.6% and a seizure-free rate of 64.1% at 12 months. The overall incidence of AEs was 24.8%, with a 12-month retention rate of 94.8%. Patients with SeLECTS exhibited an increased rate of seizure freedom. For clinical practice, an initial PER dose of 1–2 mg/day at bedtime with slow titration to a maintenance dose of ≥4 mg/day is recommended. Patients receiving 4 mg/day achieved the highest seizure-free rate,thus the optimal maintenance dose of PER should be individualized based on clinical response and patient tolerability. In conclusion, PER is an effective and safe initial monotherapy for this population, especially for patients with SeLECTS. Future investigations should focus on high-quality prospective studies with expanded sample sizes to further confirm the efficacy and safety of PER.

## Data Availability

The original contributions presented in the study are included in the article/Supplementary Material, further inquiries can be directed to the corresponding author.
