# Supplementary material for: Efficacy and safety of perampanel as the first medication for children with newly diagnosed epilepsy: a real-world single-center prospective observational study
Source: Front Pediatr. 2026 May 28;14:1767763. doi: 10.3389/fped.2026.1767763 (PMC13254166; doi:10.3389/fped.2026.1767763)
Supplement: Supplementary file 1 [file Datasheet1.pdf]

Supplementary Table 1. Clinical Characteristics of Patients with Seizure Worsening (n=5)

Patient	Age (y) / Sex	Etiology	Number of seizures during the 3 months before enrollment	PER Maintenance Dose (mg/d)	Seizure Type
1	12/F	Unknown	3	4	Focal-onset
2	10/M	Genetic	13	4	Focal-onset
3	12/M	Unknown	2	6	Focal-onset
4	9/M	Unknown	20	2	Generalized-onset
5	10/F	Unknown	10	2	Generalized-onset

M, male; F, female;
